# ACTION-IO as a platform to understand differences in perceptions, attitudes, and behaviors of people with obesity and physicians across countries – the Israeli experience

**DOI:** 10.1186/s13584-020-00404-2

**Published:** 2020-10-21

**Authors:** Dror Dicker, Batya Kornboim, Rakefet Bachrach, Naim Shehadeh, Shani Potesman-Yona, Gabriella Segal-Lieberman

**Affiliations:** 1 Department of Internal Medicine D & obesity clinic, Hasharon Hospital, Rabin Medical Center, Petah Tikva, Sackler School of Medicine, Tel Aviv University, Keren Kayemet St. 7, 49100 Petah Tikva, Israel; 2 Department of Family Medicine, Clalit Health Services, Haifa, Israel; 3 Department of Family Medicine, Clalit Health Services, Nethania, Israel; 4 Endocrinology, Diabetes and Metabolism Institute, Rambam Medical Center, Haifa, Israel; 5 Novo Nordisk, Kfar-Saba, Israel; 6 Center for Weight Management, Institute of Endocrinology, Sheba Medical Center, Ramat-Gan, Sackler School of Medicine, Tel Aviv University, Tel Aviv, Israel

**Keywords:** ACTION-IO, Obesity, Perceptions, Barriers, Israel

## Abstract

**Background:**

Obesity is a highly prevalent, complex, and chronic relapsing disease with a considerable unmet medical need. We aimed to identify perceptions, attitudes, behaviors, and barriers to effective obesity treatment among people with obesity (PwO) and physicians in Israel.

**Methods:**

The ACTION-IO study was an online survey conducted in 11 countries, including Israel. Findings from the Israeli cohort are reported here. Israeli respondents were PwO (body mass index of ≥30 kg/m^2^ based on self-reported height and weight) and physicians primarily in direct patient care.

**Results:**

In total, 750 PwO and 169 physicians completed the survey in Israel. Although most PwO (70%) and physicians (95%) perceived obesity as a chronic disease, the majority of PwO assumed full responsibility for their own weight loss (88%) compared with only 19% of physicians who placed the responsibility for weight loss on their patients with obesity. Many PwO (62%) and physicians (73%) agreed that a complete change in lifestyle would be required for PwO to lose weight and felt that treatment of obesity should be a team effort between different healthcare professionals (HCPs; 80 and 90%, respectively). Dietitians were considered by 82% of physicians to be the most effective professionals in helping PwO achieve their weight loss goals. Many PwO (69%) liked that their HCP initiated weight management discussions and 68% of those who had not previously discussed their weight would like their HCP to initiate the conversation. However, among PwO who had discussed their weight with an HCP, 59% considered the discussions to be a little helpful or not at all helpful. The beliefs that patients have little interest in or motivation for losing weight were identified by physicians as the main reasons (71 and 70%, respectively) for not initiating weight management discussions.

**Conclusions:**

In line with the ACTION-IO international study, our Israeli dataset reveals a need to improve awareness, primarily among physicians, on the physiologic basis and clinical management of obesity, including how to approach weight and weight management discussions during patient consultations.

**Trial registration:**

Registered at ClinicalTrials.gov, NCT03584191. Data first posted on ClinicalTrials.gov: 12 July 2018 - ‘Retrospectively registered’.

## Background

Obesity is a chronic relapsing disease with a high disease burden that is due to the associated metabolic, mechanical, malignant, mental, and monetary complications [1, 2]. In Israel, 26.1% of adults and 11.9% of children have obesity [3]. Due to the high disease burden of obesity, the Israeli Association for the Study of Obesity, endorsed by the Israeli Medical Association, classified obesity as a disease in May 2018 [4].

To tackle the obesity epidemic, the Israeli Ministry of Health has issued several preventive measures including a healthy eating campaign, guidance on teaching healthy nutrition in schools, and most recently a nationwide food labeling program [5]. Healthcare provision in Israel is managed by four Health Maintenance Organizations (HMO) [6], which have established an educated and effective primary physician care service. The HMOs offer the following options for people with obesity (PwO): dietitian counseling; lifestyle coaching; bariatric surgery; and pharmacotherapy, including phentermine, orlistat, liraglutide, and lorcaserin. However, pharmacologic interventions are not currently reimbursed in the national health basket for the treatment of obesity. Further improvements to obesity care require a deeper understanding of the disease itself and identification of the gaps between current and optimal management of obesity.

The Awareness, Care, and Treatment In Obesity maNagement International Observation (ACTION-IO) study aimed to identify the perceptions, attitudes, and behaviors of PwO and physicians and assess potential barriers to effective obesity care [7]. The aim of this analysis was to compare the National Israeli and Global datasets to determine the common or distinct perceptions, attitudes, and behaviors of PwO and physicians, which could aide in building an ACTION plan for the future treatment of obesity in Israel.

## Methods

Methodology for the ACTION-IO study has been reported previously [7]; it was a cross-sectional, non-interventional, descriptive study that collected data by an online survey in Australia, Chile, Israel, Italy, Japan, Mexico, Saudi Arabia, South Korea, Spain, the United Kingdom (UK), and the United Arab Emirates (UAE). The survey was conducted by a third-party vendor (KJT Group [Honeoye Falls, NY, USA]); Israeli responses were collected between August 27, 2018 and October 22, 2018. The ACTION-IO study was designed by the study steering committee (including medical doctors employed by Novo Nordisk), with support from KJT Group, and based on ACTION US [8] and ACTION Canada [9]. To avoid bias, questionnaire items were carefully phrased and presented in the same order for each respondent and items in a list were displayed alphabetically, categorically, chronologically or randomly, as relevant for each response set. For PwO survey questions regarding weight management conversations and outcomes, the term ‘healthcare professional’ (HCP) was used and included physicians, specialists, dietitians (non-physicians), pharmacists, nurses, or diabetes educators. All other PwO survey questions used the term ‘physician’. Data collection and analysis was undertaken by KJT Group. A local ethics committee/independent review board approved the questionnaires. The study was conducted in accordance with the Guidelines for Good Pharmacoepidemiology Practices [10] and is registered with ClinicalTrials.gov, number NCT03584191.

Eligible Israeli PwO were 18 years or older with a current body mass index (BMI; based on self-reported height and weight) of at least 30 kg/m^2^. PwO were excluded if they declined to provide income, were pregnant, participated in intense fitness or body building programs, or had significant, unintentional weight loss in the past 6 months. Eligible Israeli physicians were medical practitioners, in practice for 2 years or more, with at least 50% of their time spent in direct patient care, and who had seen 100 or more patients in the past month, at least 10 of whom had a BMI of at least 30 kg/m^2^. Physicians specializing in general, plastic, or bariatric surgery were excluded. All respondents provided electronic informed consent prior to initiation of the screening questions and survey.

De-identified data were analyzed by KJT Group using SPSS (IBM, version 23.0), Stata (StataCorp LLC, version IC 14.2), and Excel (Microsoft, version 2016). Data were summarized using descriptive statistics (means, medians, and frequencies) and tests of differences (chi squared, t-tests), where appropriate. Only data from those who completed the survey were included in the main analyses. A sub-analysis of the available demographic and characteristic data was conducted and included respondents who suspended within the initial screening questionnaire or main survey. An additional subgroup analysis of Israeli PwO who had achieved a weight loss of at least 5% body mass in the past 3 years and had maintained the weight loss for ≥1 year (i.e., maintained 5% weight loss) vs PwO who had not achieved a weight loss of at least 5% body mass in the past 3 years or had not maintained the weight loss for ≥1 year (i.e., did not maintain 5% weight loss) was performed. Statistical significance testing was conducted for relevant analyses using two-tailed chi square tests, t-tests, or z-tests and a significance threshold of *p* < 0.05. Adjustment for multiple testing was not undertaken as this research was exploratory and descriptive in nature. Respondents were recruited via an online panel company, to whom they had provided permission to be contacted for research purposes. All Israeli respondents were recruited through email where possible; physicians were also recruited by telephone or in-person.

To reduce sampling bias and ensure that the group was largely representative of the Israeli population, a stratified sampling approach was used for PwO, whereby the outbound sample was sent according to pre-determined demographic targets based on gender, age, household income, education and region. Targets were established based on data from the Organization for Economic Co-operation and Development (Labour Force Survey, 2018) and the US Census Bureau, International Data Base, and were monitored throughout data collection to ensure population representativeness. A set of screening questions were used to determine eligibility based on these demographic targets; subsequently, only those who had a BMI of at least 30 kg/m^2^ (based on self-reported height and weight), and who met the other eligibility criteria detailed above, were permitted to complete the full survey. In addition, the final PwO sample, including those failing to qualify for the survey, was subsequently weighted to representative demographic targets within each country for age, gender, household income, education, and region. The physician data were not weighted. The sub-analysis of the available demographic and characteristic data included data from respondents who completed the survey and from those who suspended within the screening questionnaire or main survey; all data were unweighted.

## Results

### Perception of obesity as a chronic disease

A total of 750 PwO and 169 physicians in Israel completed the survey (Table 1). The response rates for PwO and physicians were 63 and 21%, respectively; the eligibility rates were 14 and 65%, respectively. Of the physicians, 66% considered themselves to be obesity specialists. The demographics and characteristics of PwO or physicians who completed the survey were generally comparable with those of PwO or physicians who suspended either the screening questionnaire or main survey (Table S1, Additional file 1). However, some differences were observed for PwO according to BMI classification. A significantly higher proportion of PwO with Class I obesity (30–34.9 kg/m^2^) suspended during the screening questionnaire or main survey when compared with those who completed both (77% vs 65%, respectively; *p* = 0.05). In contrast, fewer PwO with Class II (35–39.9 kg/m^2^) or Class III (≥40 kg/m^2^) obesity suspended during the screening questionnaire or main survey (18 and 5%, respectively) when compared with those who completed both (23 and 12%, respectively; *p* = 0.05 for PwO with Class III obesity).

**Table 1:** Sample demographics and characteristics

	PwO (***n*** = 750)	Physicians (***n*** = 169)
Age, years (range)	43 (18–83)	54 (33–74)
Gender, n (%)		
Male	260 (35)	79 (47)
Female	490 (65)	90 (53)
Other	0	0
BMI classification, n (%)		
Respondents	750 (100)	122 (72)^a^
Underweight or healthy range (< 25 kg/m^2^)	–	57 (47)
Overweight (25–29.9 kg/m^2^)	–	50 (41)
Obesity Class I (30–34.9 kg/m^2^)	489 (65)	10 (8)
Obesity Class II (35–39.9 kg/m^2^)	171 (23)	3 (2)
Obesity Class III (≥40 kg/m^2^)	90 (12)	2 (2)
Number of comorbidities, n (%)		
0	225 (26)	–
1	180 (21)	–
2	139 (21)	–
3	100 (16)	–
≥ 4	106 (15)	–
Physician category, n (%)		
PCP	–	101 (60)
Specialist	–	68 (40)
Endocrinologist	–	34 (20)
Gastroenterologist	–	20 (12)
Internal medicine (non-PCP)	–	10 (6)
Other	–	4 (2)
Obesity specialist, n (%)^b^		
Yes	–	112 (66)
No	–	57 (34)

Abbreviations: BMI, body mass index; PCP, primary care physician; PwO, people with obesity

All PwO N numbers are from unweighted data. All PwO percentages for demographic results (age, gender) are also from unweighted data, whereas, the PwO percentages for non-demographic results are from weighted data. Physician data were not weighted, therefore, all physician N numbers and percentages are from unweighted data.

^a^A total of 47 physicians declined to provide their weight and/or height measurements; BMI classification was not determined for these respondents

^b^A physician who meets at least one of the following criteria: at least 50% of their patients are seen for obesity/weight management; has advanced/formal training in the treatment of obesity/weight management beyond medical school; considers themselves to be an expert in obesity/weight loss management; or works in an obesity service clinic

**Fig. 1:**
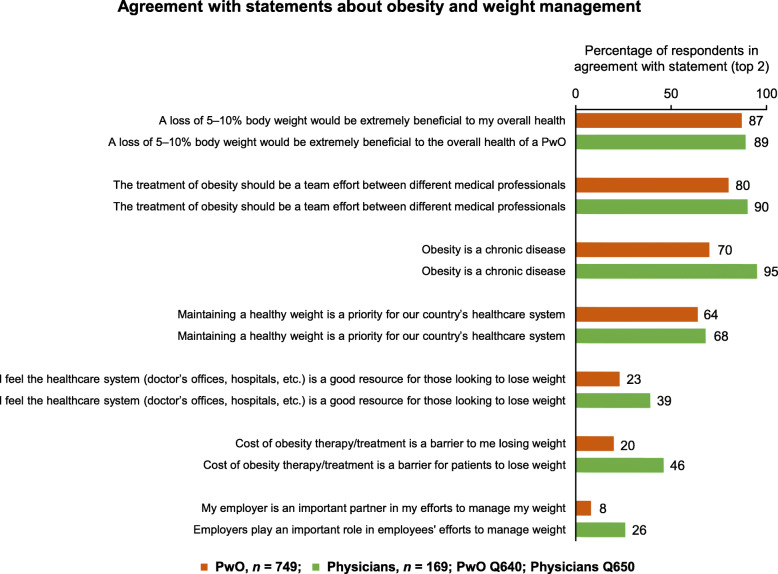
PwO and physician agreement with statements about obesity and weight management. Rated on a scale of 1–5. Physicians = green; PwO = orange. *n* size for PwO is less than total due to respondents selecting not sure for attributes. Abbreviation: PwO, people with obesity

The majority of PwO (70%) and physicians (95%) agreed with the statement that obesity is a chronic disease (Fig. 1, item 3). A total of 75% of PwO and 69% of physicians believed that obesity has a large impact on overall health; this compares with 69–87% of PwO and 78–88% of physicians for stroke, cancer, or chronic obstructive pulmonary disease (Fig. S1, Additional file 1). There was a broad understanding that the treatment of obesity should be a team effort between different medical professionals (PwO 80%, physicians 90%); however, a lack of trust in the healthcare system as a good resource for weight loss was common among PwO (Fig. 1, item 5). Cost of obesity therapy/treatment was not considered to be a significant barrier in Israel (Fig. 1, item 6).

### Weight loss barriers

**Fig. 2:**
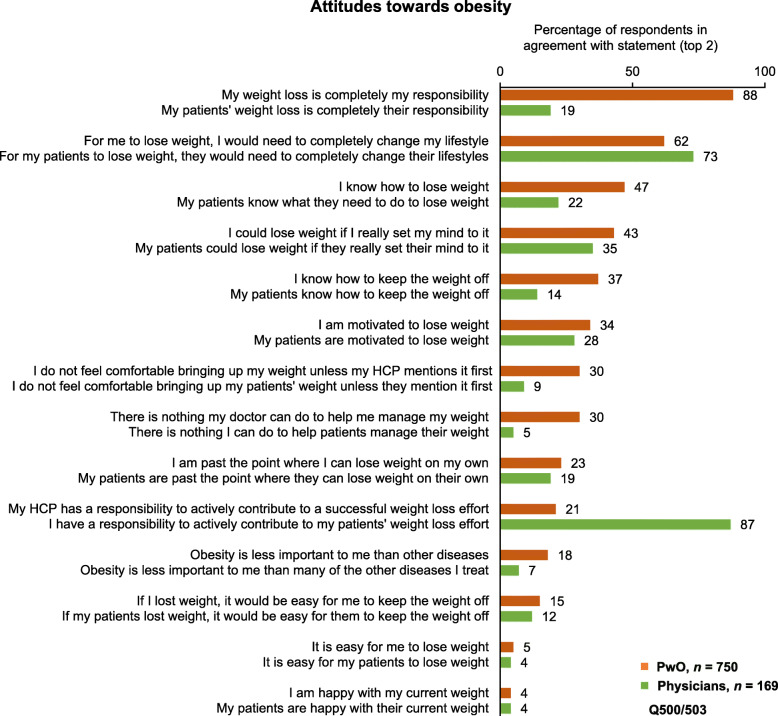
PwO and physician agreement with statements regarding attitudes towards obesity. Rated on a scale of 1–5. Physicians = green; PwO = orange. Abbreviations: HCP, healthcare professional; PwO, people with obesity

Although PwO recognized obesity as a chronic disease, the majority assumed full responsibility for weight loss (88%) and largely attributed struggles with obesity to lifestyle factors (62%; Fig. 2, items 1–2). Conversely, only 19% of physicians considered weight loss the sole responsibility of PwO. Whilst 73% of physicians agreed that a complete change in lifestyle would be required for PwO to lose weight, 87% recognized that HCPs needed to contribute to their patients’ weight loss efforts (Fig. 2, items 1, 2, and 10); less than a third (28%) of physicians agreed that their patients were motivated to lose weight (Fig. 2, item 6). PwO and physicians considered unhealthy eating habits (PwO 63%, physicians 89%) and lack of exercise (PwO 73%, physicians 84%) as barriers to weight loss; in comparison, only 49% of PwO and physicians considered the genetic factors underlying obesity to be a barrier (Fig. S2, Additional file 1).

### Weight loss attempts and outcomes

**Fig. 3:**
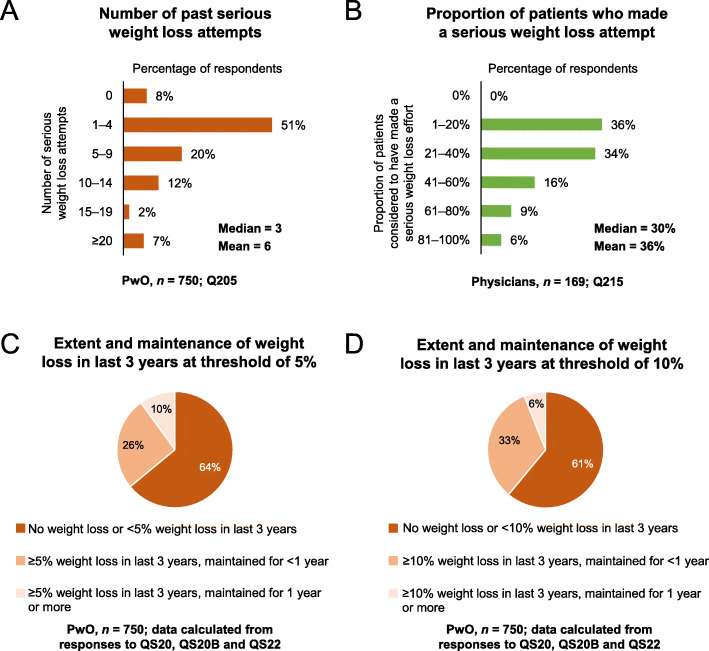
Weight loss efforts and response to intervention. **a** Number of past serious weight loss attempts (PwO). **b** Proportion of patients considered to have made a serious weight loss attempt reported by physicians. **c**, **d** PwO extent and maintenance of weight loss in last 3 years at threshold of (**c**) 5% or (**d**) 10% of total body weight. Physicians = green; PwO = orange. Abbreviation: PwO, people with obesity

Most PwO (92%) had made a serious effort to lose weight (e.g., followed a program, set goals, put their mind to it, or worked with a qualified HCP) on at least one occasion, with a mean number of 6 attempts (Fig. 3a). In contrast, physicians reported that only 36% of their patients with obesity had made a serious weight loss effort (Fig. 3b); of these, physicians considered that 22% had successfully responded (Fig. S3, Additional file 1). In general, PwO struggled to lose weight and to maintain any weight loss (Fig. 3c, d): weight loss of at least 5% body mass over the past 3 years was reported by only 38% of PwO; of those, only 26% were able to maintain the weight loss for at least 1 year (10% of PwO total; Fig. 3c). Furthermore, only 6% of PwO were able to maintain a weight loss of at least 10% body mass for 1 year or more (Fig. 3d). Interestingly, a higher proportion of PwO who had maintained a weight loss of at least 5% body mass had made a serious effort to lose weight on at least 5–9 or 10–14 occasions (34 and 22%, respectively), when compared with PwO who had not maintained the weight loss (18 and 11%, respectively). Of PwO who could not maintain the weight loss, 50% cited no longer following their eating plan as one of the main reasons for their weight regain.

### Weight management conversations and outcomes

**Fig. 4:**
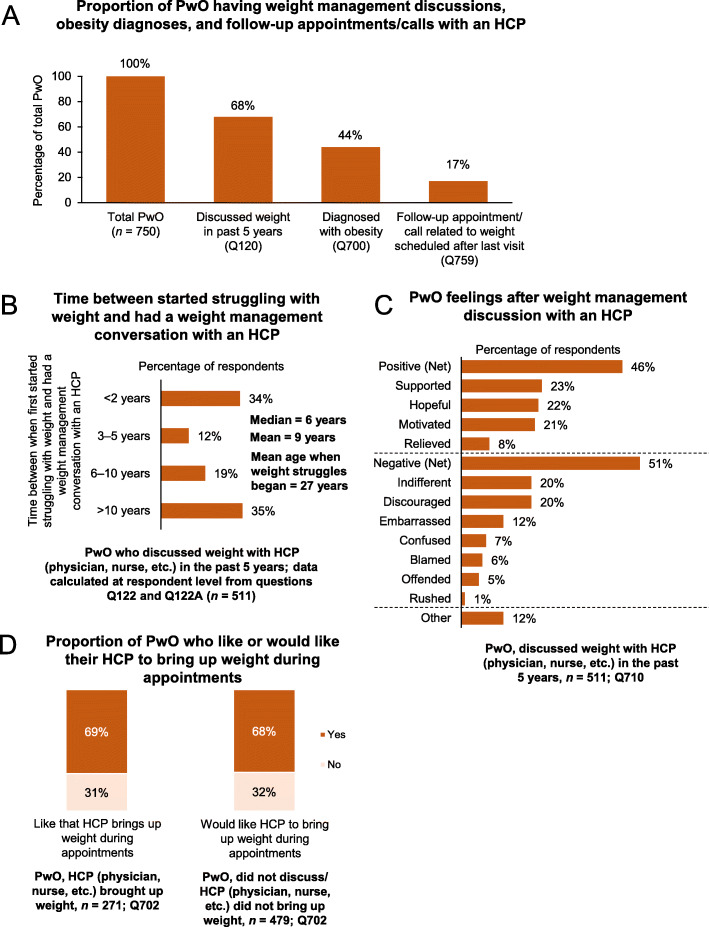
Weight management conversations and outcomes. **a** Proportion of PwO having weight management discussions with an HCP, obesity diagnoses and follow-up appointments/calls. **b** Of PwO who had discussed their weight with an HCP in the past 5 years, proportion who had the discussion less than 2 years, 3–5 years, 6–10 years, or more than 10 years after they first started struggling with their weight. **c** PwO feelings after discussing their weight with an HCP. **d** Proportion of PwO who like or would like their HCP to bring up weight during appointments. Abbreviations: HCP, healthcare professional; PwO, people with obesity

Around two-thirds of PwO had discussed their weight with an HCP (physician, nurse, etc.) in the past 5 years; of these, only 44% were diagnosed with obesity and only 17% had a follow-up appointment or call related to weight management scheduled (Fig. 4a). Despite only 9% of physicians stating that they did not feel comfortable bringing up weight loss unless it was mentioned by the patient first (Fig. 2; 12% internationally [7]), weight management discussions between PwO and HCPs took place a mean 9 years after the PwO first started struggling with excess weight or obesity; a time delay of more than 10 years between weight struggles and weight management discussions was reported in 35% of PwO (Fig. 4b; 17% internationally [7]). According to PwO, weight management discussions were initiated by the patient in 47% of cases (32% of PwO total; Fig. S4, Additional file 1). Physicians cited obesity-related complications as the main reason for initiating a weight management conversation (Fig. S5, Additional file 1).

Discussions between PwO and HCPs were considered to be a little helpful or not at all helpful by 59% of PwO who had discussed their weight with an HCP during the past 5 years (Fig. S6, Additional file 1). Moreover, around half of PwO (51%) had net negative feelings following their most recent weight management discussion with their HCP, of which 5% reported feeling offended (Fig. 4c). Interestingly, the mean weight of PwO who experienced negative feelings following a weight management conversation was slightly higher when compared with those who did not have any negative feelings (99.7 kg and 97.2 kg, respectively). Subtle directional differences were also observed when stratifying patients according to their BMI; while the proportion of PwO with Class I obesity (BMI 30–34.9 kg/m^2^) who had negative feelings (i.e., yes) vs those who did not have any negative feelings (i.e., no) did not differ (yes, 50%; no, 50%), PwO with Class II (BMI 35–39.9 kg/m^2^) or Class III (BMI ≥40 kg/m^2^) obesity were generally more likely to have had negative feelings after weight management discussions with their HCP (yes, 53%; no, 47% for each BMI class). In addition, fewer PwO who were unable to maintain a weight loss of at least 5% body mass than those who had successfully maintained their weight loss felt supported (22% vs 36%), motivated (20% vs 33%), or hopeful (21% vs 31%) after weight management discussions with their HCP. However, a high proportion (69%) of PwO liked that their HCP brought up the subject of weight during appointments and 68% of PwO who had not had a weight management discussion previously would like their HCP to initiate the topic (Fig. 4d). General improvements in eating habits and physical activity levels were weight management methods frequently discussed between PwO and HCPs (Fig. S7A, Additional file 1). Interestingly, some directional differences were observed when stratifying PwO according to weight loss outcomes; weight loss/bariatric surgery was frequently discussed with a significantly higher proportion of PwO who had maintained a weight loss of at least 5% body mass (30%) vs PwO who had not maintained their weight loss (11%). In contrast, visiting a dietitian was frequently discussed with a higher proportion of PwO who were unable to maintain a weight loss of at least 5% body mass (57%), when compared with PwO who had maintained their weight loss (47%).

### Barriers to weight loss conversations

**Fig. 5:**
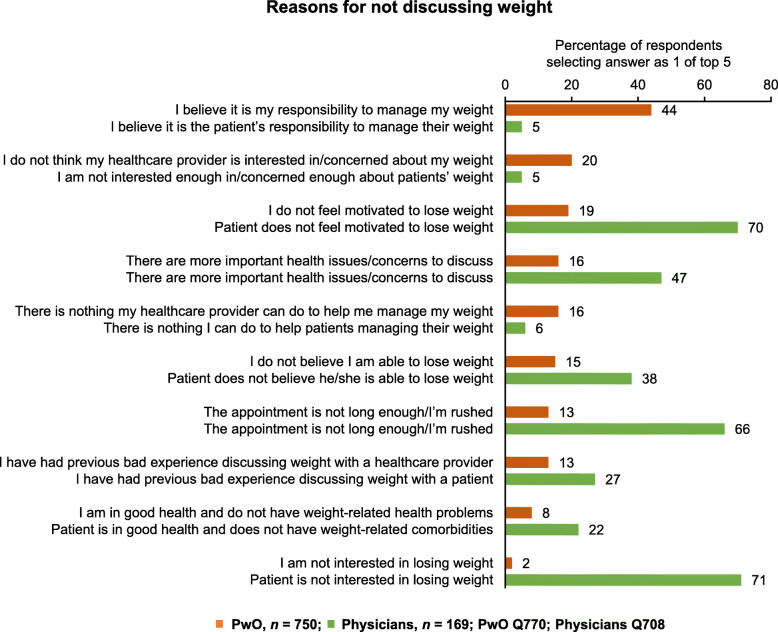
Reasons for not discussing weight with an HCP or patient, with at least 10% difference. Reasons for not discussing weight with an HCP (PwO responses) or patient (physician responses) with at least 10% difference between PwO and physicians. See Supplementary Figure S1 (Additional file 1) for all reasons. Physicians = green; PwO = orange. Abbreviations: HCP, healthcare professional; PwO, people with obesity

The most common reason PwO gave for not discussing weight management with an HCP was a belief that it was their responsibility to manage their own weight (PwO 44%, physicians 5%; Fig. 5 and Fig. S8, Additional file 1). In contrast, physicians gave patient disinterest (Israel 71%, international cohort 71% [7]) and lack of patient motivation (Israel 70%, international cohort 68% [7]) as the main reasons for not discussing weight management; disinterest and lack of motivation were given as reasons by only 2 and 19% of PwO, respectively (Fig. 5; international cohort 7 and 20%, respectively [7]). Limited appointment time was also cited as a factor in not initiating weight loss conversations by 66% of physicians (Fig. 5). Of PwO who had not discussed weight with an HCP, 56% would consider discussing their weight with a dietitian (Fig. S9, Additional file 1).

### Perceived effectiveness of weight management methods

Among PwO and physicians, general improvements in eating habits (65 and 84%), bariatric surgery (63 and 69%), and increasing physical activity levels (60 and 72%) were perceived as effective weight management methods (Fig. S7B, Additional file 1). In contrast, exercise tracking (23 and 42%), over-the-counter weight loss medications (21 and 10%), visiting an obesity specialist (16 and 32%), and sleep quality management (6 and 29%) were all considered less effective weight management methods (Fig. S7B, Additional file 1). Interestingly, prescription weight loss medications were mainly perceived as having low efficacy by PwO when compared with physicians (27% vs 40%, respectively; Fig. S7B, Additional file 1).

### Perceived effectiveness of HCPs in supporting PwO

Dietitians (non-physicians) were considered by 82% of physicians to be the most effective professionals in helping patients with obesity (Fig. S10, Additional file 1). A high proportion of physicians (20% vs 15% internationally; ACTION-IO study steering committee, personal communication) also believed in the role of surgeons in helping PwO; referrals to other specialists, such as cardiologists, were less frequently recommended (Fig. S10, Additional file 1).

## Discussion

The Israeli Medical Association [4] recognizes obesity as a chronic disease, thereby joining other international and national medical organizations including the World Obesity Federation [1], the European Association for the Study of Obesity [11], The Obesity Society [12], the American Medical Association [13], and the UK Royal College of Physicians [14]. Genetic factors have been shown to play a role in predisposition to obesity and multiple pathophysiologic mechanisms are associated with obesity disease development [1, 15]. Additionally, obesity has recently been defined as an adiposity-based chronic disease (ABCD), with obesity-related complications mainly being attributed to abnormal physical forces (fat mass disease) and disturbed endocrine and immune responses (sick fat disease) [16]. Despite this, 88% of PwO from Israel believed that weight loss was completely their own responsibility and only 21% agreed that their HCP should actively contribute to their effort to lose weight. This is similar to the international results, in which 81% of PwO thought that weight loss was their sole responsibility and 26% agreed that HCPs had a responsibility to contribute to their weight loss [7]. This feeling of self-responsibility was the reason why 44% of PwO, both internationally [7] and from Israel, did not have a weight management conversation with an HCP (physician, nurse, etc.). This underscores the need for widespread education on the genetic and pathophysiologic processes underlying obesity disease to remove the barrier of patient feelings of self-blame and to facilitate open conversations between PwO and physicians. Educating PwO on the various weight management methods and treatment options available may also encourage them to initiate a weight management discussion with their physician. Additionally, PwO motivation to lose weight was lower in Israel compared with the international results; 34% of PwO in Israel vs 48% internationally [7] felt motivated to lose weight, which may be linked to feelings of self-blame, multiple failed weight loss attempts in the past, or a lack of knowledge concerning treatment options.

Data from this study suggest that the majority of PwO, both internationally and in Israel, would like HCPs to address their weight during appointments. Physicians in Israel were confident in initiating weight loss conversations, with only 9% stating that they do not feel comfortable bringing up weight loss unless this is mentioned by the patient (12% internationally [7]). However, in contrast with the finding that 64% of international PwO had positive feelings following a weight management discussion [7], 51% of PwO in Israel who discussed their weight in an appointment with an HCP left the conversation with a negative feeling. Interestingly, a slightly higher proportion of PwO with Class II or Class III obesity experienced negative feelings following such a conversation. Considering these findings, there is an urgent need to raise awareness among Israeli physicians regarding the correct approach to discussing weight and weight management with PwO. Additionally, there is a need to understand the potential challenges physicians experience during patient dialogue. Slight differences in wording have been demonstrated to have a significant impact on the physician–patient relationship; joint discussion and deduction of obesity through BMI calculation is a more effective method of conversation than the physician questioning or stating that a patient has overweight [17]. Furthermore, there is a disconnect between the actual and perceived attitudes of PwO both internationally and in Israel as to the reasons for not initiating weight loss conversations. International physicians [7] and those in Israel cited low patient motivation and patient disinterest in losing weight (all ~ 70%) as the main reasons for not initiating weight loss conversations; in contrast, low motivation and disinterest were factors for only ~ 20 and < 10% of PwO, respectively, both internationally [7] and in Israel. Multiple studies have reported obesity stigmatization by HCPs, including viewing PwO as having low self-discipline [18]. Together with the data presented here, this reflects a need for increasing awareness among physicians on attitudes towards obesity.

Adopting a multidisciplinary approach to obesity care, involving physicians, nurses and/or nurse practitioners, dietitians and psychologists, may facilitate the clinical management of obesity and help address the needs of PwO. Indeed, most Israeli PwO and physicians felt that treatment of obesity should be a team effort between different medical professionals (80 and 90%, respectively) and agreed that a complete change in lifestyle would be required for PwO to lose weight (62 and 73%, respectively). In line with these findings, the results presented here suggest that physicians in Israel defer responsibility to dietitians when treating PwO, with most physicians (82%) believing that dietitians are most effective in helping PwO to achieve their weight loss goals. Physician referral and recommendation for dietary counseling has previously been shown to have a strong impact on PwO adherence to nutritional counseling [19]; therefore, active encouragement from Israeli physicians and early referral to a dietitian may lead to improved patient outcomes. A higher proportion of physicians in Israel than in the international dataset (20% vs 15%; ACTION-IO study steering committee, personal communication) also believed in the role of surgeons in helping to treat PwO, which may account for the high proportion of bariatric surgery in Israel [20].

The time delay between when PwO first began to struggle with their weight and had a weight management conversation with their HCP has been identified as a potential barrier to effective obesity care [7]. In Israel, the most common reason for PwO not discussing their condition with an HCP was the belief that losing weight was entirely their own responsibility. On the other hand, physicians considered patient disinterest and lack of patient motivation as the main reasons for not initiating a conversation with their patients. These differing perceptions may explain the long duration between weight struggle onset and HCP conversation of over 10 years in 35% of PwO compared with 17% internationally [7]. Cultural differences may also prevent earlier initiation of weight management conversations. Reducing the time delay to HCP intervention may reduce associated disease complications and lessen the disease burden, highlighting the need for early intervention prior to the development of complications. Improvements in accessibility to healthcare information and obesity services in Israel are also required and may be achieved through development of specialist obesity units and group educational resources. Overall, cost of obesity therapy/treatment was not considered to be a significant barrier in Israel; however, the nature of this study may mask regional and socioeconomic differences.

A summary of policy recommendations is provided in Table 2 and outlines the multimodal approach required to have a substantial effect on accelerating obesity prevention, improving awareness, providing tailored training and clinical management of obesity. We suggest that each recommendation should not be viewed in linear form. The implementation of each strategy influences the success of the others and has the potential for combined impacts that can further accelerate progress in preventing, managing, and treating obesity.

**Table 2:** Summary of recommendations

Organizational body	Action	Outcome measures	Supporting Israeli ACTION-IO data
**Government**	• Acknowledge obesity as a disease	• Gain recognition as a significant public health hazard • Diagnosis may increase referral rates to specialists and/or follow-up appointments	• 70% of PwO and 95% of HCPs agreed that obesity is a chronic disease • 81% of PwO thought that weight loss was their sole responsibility; 44% reported this as a reason for not discussing weight with an HCP • Only 44% of PwO were diagnosed with obesity; 17% had a follow-up appointment
**Health medical organization**	• Build a network of multidisciplinary obesity treatment clinics that include obesity medicine physicians, dietitians, psychosocial services, and physical exercise counseling	• Create a multidisciplinary support system for people with obesity	• Most PwO (87%) and HCPs (83%) do not believe the healthcare system and society in general currently meet the needs of PwO • Only 23% of PwO felt that the healthcare system was a good resource for weight loss • Most PwO (80%) and HCPs (90%) felt that treatment of obesity should be a team effort between different medical professionals
	• Prioritize people with obesity for vaccination against viral infections	• Reduce the risk of complications for a high-risk group	• Approximately 3/4 of PwO have ≥1 comorbidity
	• Create a campaign for the public to promote awareness that obesity is a biological disease, not a lifestyle choice	• Educate the public on the etiology of obesity and the obesogenic environment	• Only 49% of PwO or HCPs considered the genetic factors underlying obesity to be a barrier to weight loss
**Medical schools**	• Incorporate obesity medicine teaching hours into the pre-clinical (biological and genetic basis) and the clinical (approach to treatment) years	• Reduce time gap between people struggling with excess weight and seeking medical help • Provide tailored obesity care • Increase obesity diagnosis, follow-up appointments, and referrals • Improve weight loss outcomes for PwO	• There was a mean delay of 9 years between the time PwO began struggling with excess weight or obesity and the first weight management discussion with their HCP • Among PwO (68%) who had discussed their weight with an HCP in the past 5 years, 59% considered the discussions to be a little helpful or not at all helpful • 51% of PwO had negative feelings following their most recent weight management discussion with their HCP • Misperception among HCPs that patients have little interest in or motivation for losing weight (71 and 70%, respectively) were the main reasons for HCPs not initiating weight management discussions
**Israel Association for the Study of Obesity**	• Build a fellowship program for obesity medicine, approved by the Israeli Medical Association • Establish “obesity medicine schools” for physicians and dietitians • Draft obesity management guidelines and a position paper • Work with stakeholders to promote the recognition of obesity as a disease • Work with representatives of PwO on anti-stigma campaigns		

Abbreviations: HCP, healthcare professional; PwO, people with obesity

Key limitations of this study are similar to those of the global ACTION-IO study [7], including its cross-sectional and descriptive nature, reliance on self-reported height and weight, and accuracy of respondent recall. Additional limitations specific to the Israeli dataset include the relatively short recruitment period (2 months vs 4 months) when compared with the global study [7] and the low response rate among Israeli physicians. Low response rates are typically observed with survey-based research and can affect sample representativeness. While the percentage of Israeli physicians who responded and completed the survey was small, it was generally consistent with the data reported in the global study [7]. However, a high proportion of Israeli physicians considered themselves as obesity specialists (66 and 67% internationally [7]), which may have biased their responses and affected the study outcomes. It is also important to note that despite universal insurance coverage, healthcare inequities persist among the various groups of Israeli society and are largely attributed to religious, ethnical, cultural, and lingual differences [21]. Although a higher proportion of PwO in Israel responded and completed the survey when compared with the global study (63% vs 20% internationally [7]), the study design did not allow for consideration of some of these factors during sampling or weighting of PwO data. As such, response bias among PwO cannot be ruled out and may impede the generalizability of these results.

## Conclusions

Overall, the data presented herein suggest that while PwO recognize obesity as a disease, they typically assume complete responsibility for weight management and do not place high importance on the role of physicians. There is a need for physicians to initiate earlier weight loss conversations to actively encourage patients to make lifestyle changes and to recommend referrals to specialists before obesity-related complications develop. To this end, PwO and physicians in Israel have a need to improve their knowledge regarding the biologic basis of obesity and effective ways to approach weight and weight management during consultations that improve patient engagement, empowerment, and treatment.

## Supplementary information

**Additional file 1: Table S1.** Demographics and characteristics of the respondents who suspended or completed the survey. **Figure S1.** Attitudes towards obesity. **Figure S2.** Agreement with statements about weight loss barriers. **Figure S3.** Proportion of patients who responded to weight loss effort, reported by physicians. **Figure S4.** Initiation of weight management conversations. **Figure S5.** Physician criteria for initiating weight management conversations. **Figure S6.** How helpful PwO found their conversation with an HCP (physician, nurse, etc.) about weight management. **Figure S7.** Weight management methods discussed/recommended and perceived effectiveness. **Figure S8.** Reasons for not discussing weight with an HCP (PwO responses) or patient (physician responses). **Figure S9.** Healthcare professionals with whom PwO would consider having weight management discussions. **Figure S10.** Healthcare professionals considered most effective in helping PwO.

## Data Availability

Deidentified participant data are available for this Article on a specialized SAS data platform. Datasets from Novo Nordisk will be available permanently after completion of data analyses. The study protocol and statistical analysis plan will be available according to Novo Nordisk data sharing commitments. Access to data can be made through a request proposal form and the access criteria can be found online (novonordisk-trials.com). Data will be shared with bona fide researchers submitting a research proposal requesting access to data. Data use is subject to approval by the Independent Review Board.

## Abbreviations

ABCD: Adiposity-based chronic disease
ACTION-IO: Awareness, Care, and Treatment In Obesity maNagement International Observation
BMI: Body mass index
HCP: Healthcare professional
PCP: Primary care physician
PwO: People with obesity
UK: United Kingdom
UAE: United Arab Emirates
