# Supplementary material for: ACTION-IO as a platform to understand differences in perceptions, attitudes, and behaviors of people with obesity and physicians across countries – the Israeli experience
Source: Isr J Health Policy Res. 2020 Oct 21;9:56. doi: 10.1186/s13584-020-00404-2 (PMC7579877; doi:10.1186/s13584-020-00404-2)
Supplement: Supplementary file 1 — Additional file 1: Table S1. Demographics and characteristics of the respondents who suspended or completed the survey. Figure S1. Attitudes towards obesity. Figure S2. Agreement with statements about weight loss barriers. Figure S3. Proportion of patients who responded to weight loss effort, reported by physicians. Figure S4. Initiation of weight management conversations. Figure S5. Physician criteria for initiating weight management conversations. Figure S6. How helpful PwO found their conversation with an HCP (physician, nurse, etc.) about weight management. Figure S7. Weight management methods discussed/recommended and perceived effectiveness. Figure S8. Reasons for not discussing weight with an HCP (PwO responses) or patient (physician responses). Figure S9. Healthcare professionals with whom PwO would consider having weight management discussions. Figure S10. Healthcare professionals considered most effective in helping PwO. [file 13584_2020_404_MOESM1_ESM.pdf]

Additional file 1

Supplement for: Dror Dicker, et al. ACTION-IO as a platform to understand differences in perceptions, attitudes, and behaviors of people with obesity and physicians across countries – the Israeli experience

Additional file 1

TABLE S1 Demographics and characteristics of the respondents who suspended or completed the survey.

**Israeli PwO**	**PwO, suspended survey† (*n* = 323)**	**PwO, completed survey (*n* = 750)**
Age, years [*n* = 181]	41	43
Gender, (%) [*n* = 179]		
Female	65	65
Male	35	35
BMI, kg/m^2 [*n* = 57]	34	36
BMI classification, (%)		
Obesity Class I (30–34.9 kg/m^2)‡	77	65
Obesity Class II (35–39.9 kg/m^2)	18	23
Obesity Class III (≥40 kg/m^2)‡	5	12
**Israeli physicians**	**Physicians, suspended survey§ (*n* = 155)**	**Physicians, completed survey (*n* = 169)**
Age, years [*n* = 60]	52	54
Gender, (%) [*n* = 66]		
Female	55	53
Male	45	47
Physician specialty, (%) [*n* = 57]		
Family practice	37	33
Internal medicine	21	26
Endocrinology	16	20
Gastroenterology	14	12
General practice	2	7
Other	10	2

Abbreviations: BMI, body mass index; PwO, people with obesity.
Data are presented as mean or percentage. All PwO and physician data are not weighted, therefore, all N sizes and percentages are unweighted data.
†A total of 323 PwO suspended the survey; *n* = 274 suspended within the initial screening questionnaire and *n* = 49 completed the screening questionnaire and suspended within the main survey. The N sizes for each metric are variable, depending on where respondents suspended within the screening questionnaire or main survey, and are provided in the left-hand column.
‡$P = 0.05$.
§A total of 155 physicians suspended the survey; *n* = 117 suspended within the initial screening questionnaire and *n* = 38 completed the screening questionnaire and suspended within the main survey. The N sizes for each metric are variable, depending on where respondents suspended within the screening questionnaire or main survey, and are provided in the left-hand column.

Additional file 1

FIGURE S1 Attitudes towards obesity

Thoughts on impact of different health conditions on a person's overall health. Physicians = green; PwO = orange. Abbreviations: COPD, chronic obstructive pulmonary disease; PwO, people with obesity.

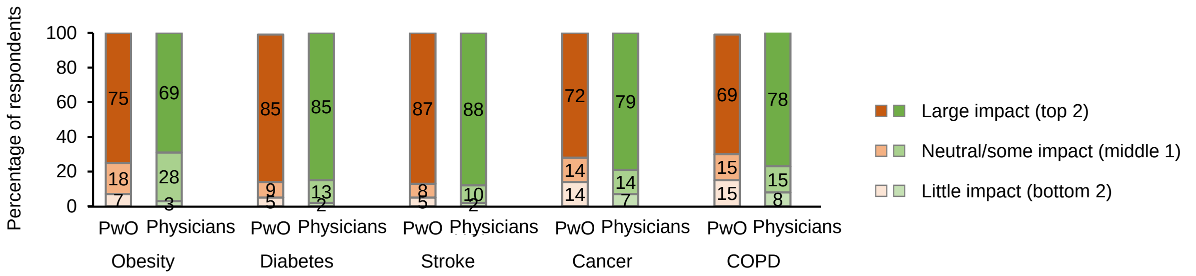

PwO familiar with condition, *n* = 750; physicians, *n* = 169; Q405; rated on a scale where 1 = very little impact, 5 = extreme impact.

Additional file 1

FIGURE S2 Agreement with statements about weight loss barriers

Physicians = green; PwO = orange. Abbreviations: PwO, people with obesity.

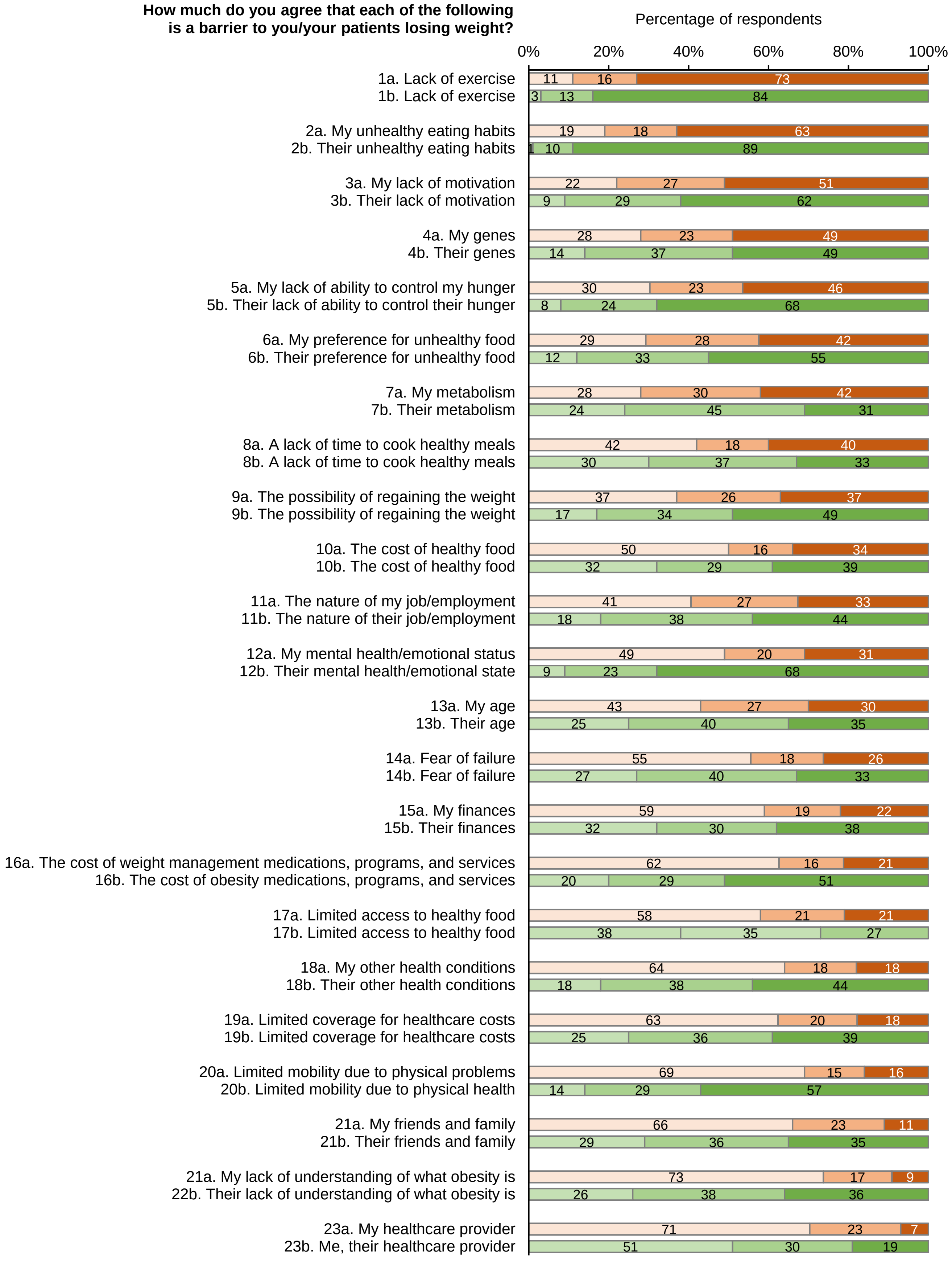

PwO, *n* = 750; Physicians, *n* = 169; Q507; rated on a scale where 1 = do not agree at all, 5 = completely agree.

Additional file 1

FIGURE S3 Proportion of patients who responded to weight loss effort, reported by physicians

Chart shows the proportion of patients who made a serious weight loss effort within the past year who were defined as successful by physicians.

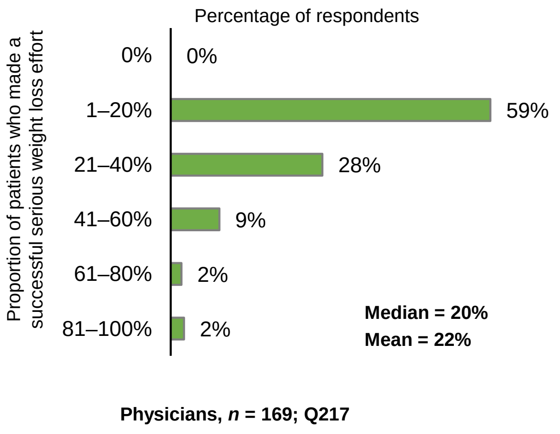

Additional file 1

FIGURE S4 Initiation of weight management conversations

(A) Proportion of PwO who discussed weight with an HCP (physician, nurse, etc.) in the past 5 years, and who initiated the conversation. (B) Proportion of patients with whom physicians discussed weight, and who initiated the conversation. Physicians = green; PwO = orange. Abbreviations: HCP, healthcare professional; PwO, people with obesity.

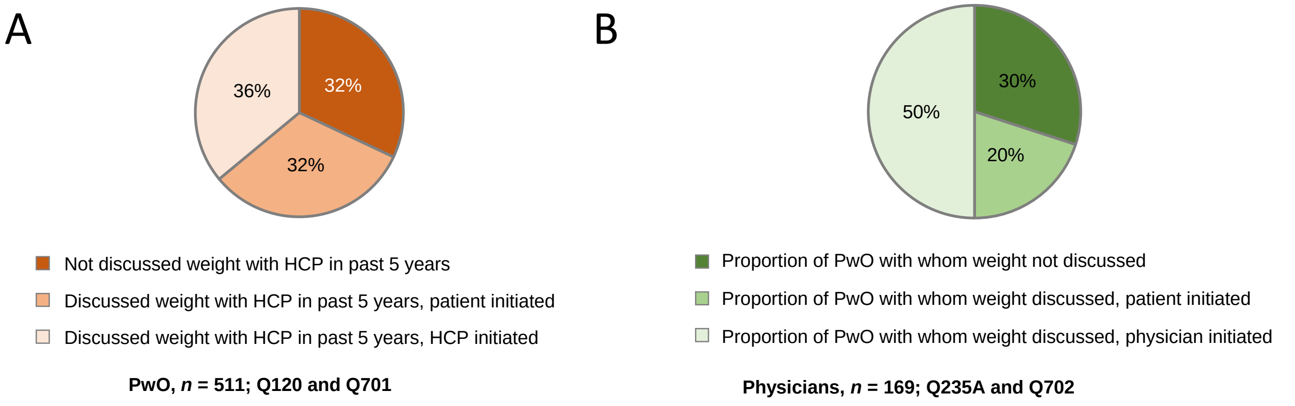

Additional file 1

FIGURE S5 Physician criteria for initiating weight management conversations

Abbreviations: BMI, body mass index.

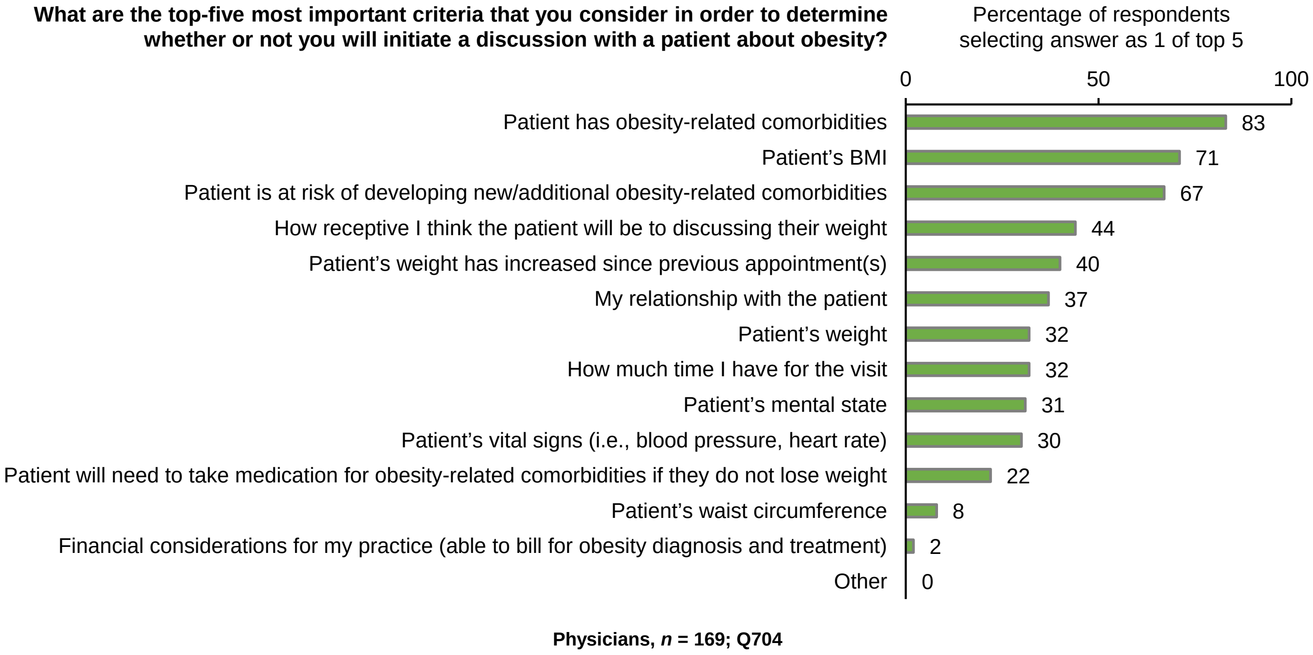

Additional file 1

FIGURE S6 How helpful PwO found their conversation with an HCP (physician, nurse, etc.) about weight management
Abbreviations: HCP, healthcare professional; PwO, people with obesity.

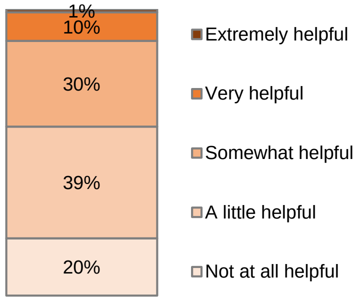

PwO, discussed weight with HCP (physician nurse, etc.) in the past 5 years, *n* = 511; Q700C

Additional file 1
FIGURE S7 Weight management methods discussed/recommended and perceived effectiveness
(A) Weight management methods discussed with an HCP reported by PwO and treatment recommended to patients reported by physicians. (B) Perceived effectiveness of weight management methods. Physicians = green; PwO = orange. Abbreviations: HCP, healthcare professional; PwO, people with obesity; QoL, quality of life.

A Discussed

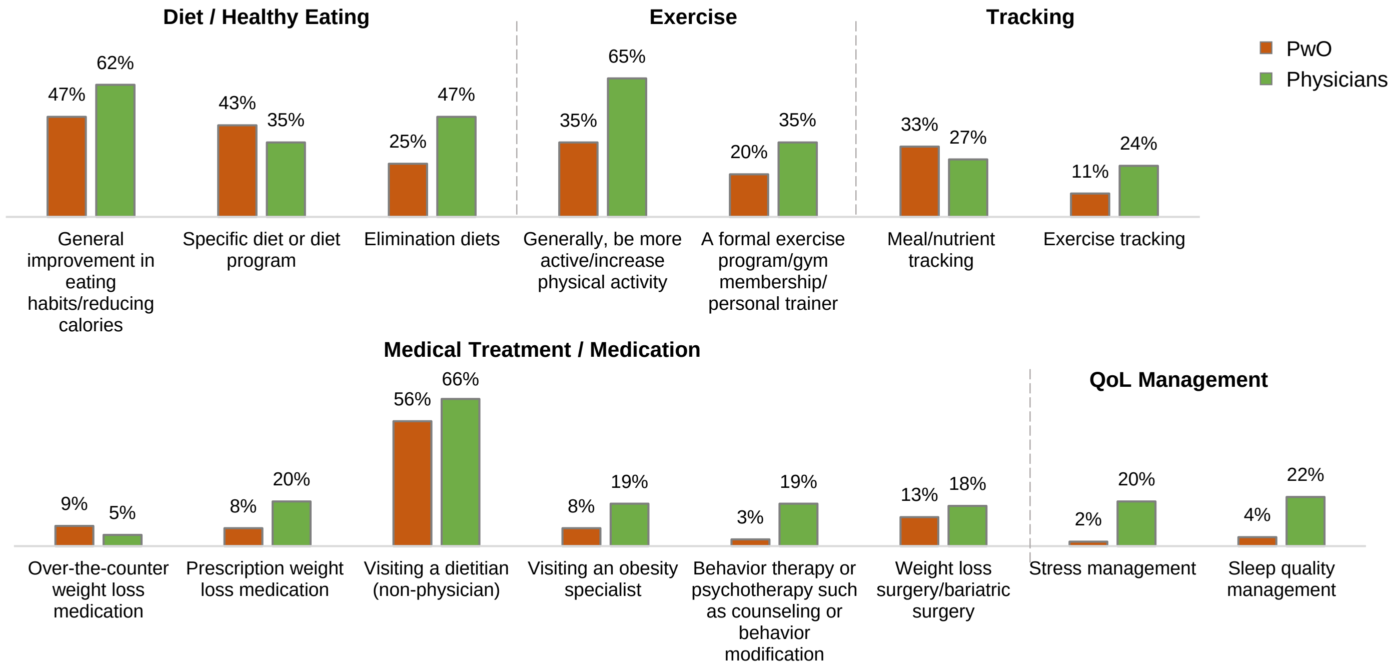

PwO, discussed with HCP and made weight loss effort, *n* = 682; Physicians, discusses weight management with patients, *n* = 165; PwO Q210A; Physicians Q128; 'Other' = 3% PwO, 4% physicians; 'none of the above' = 13% PwO, 1<% physicians

B Perceived as effective

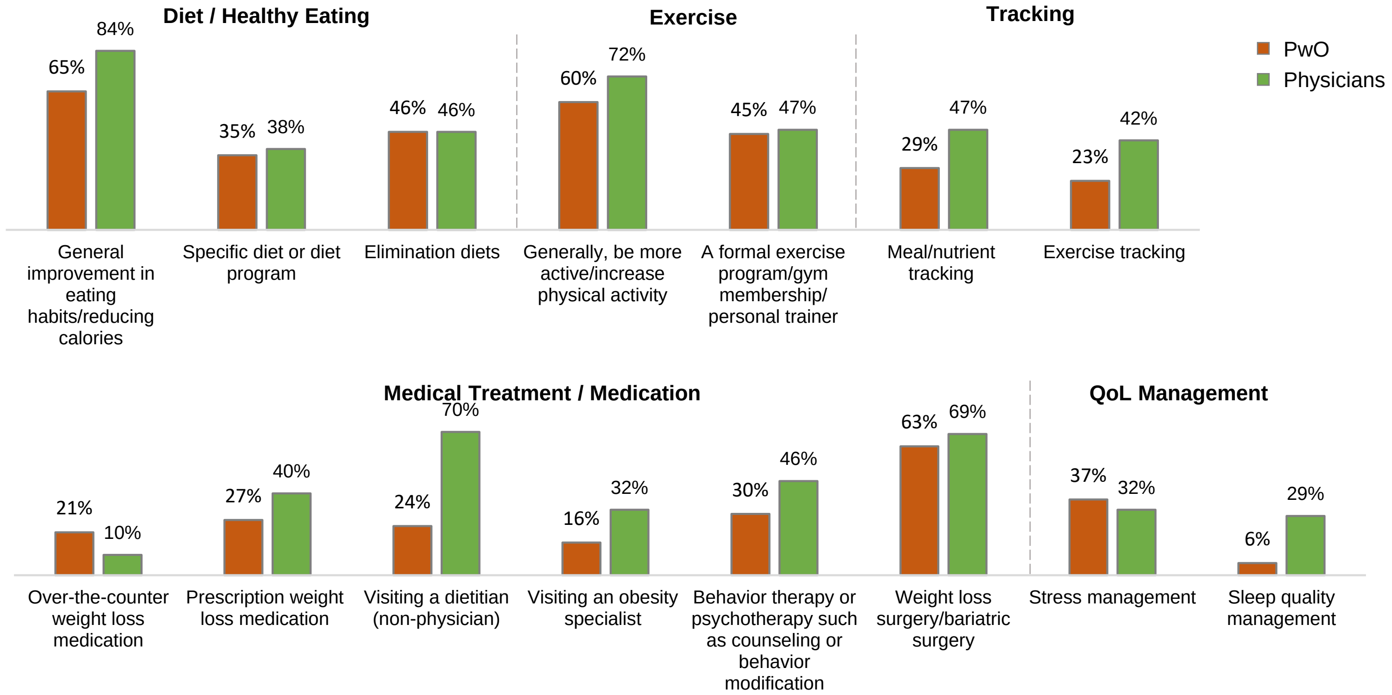

PwO, discussed with HCP, made weight loss effort, and tried method, *n* = variable base; Physicians, discusses weight management with patients, *n* = 165; PwO Q210D; Physicians Q515; 'Other' = 6% PwO, 4% physicians; 'I have not found any of these methods to be effective for weight loss'/'none of the above' = 20% PwO, 0% physicians.

Additional file 1

FIGURE S8 Reasons for not discussing weight with an HCP (PwO responses) or patient (physician responses) physicians = green; PwO = orange. Abbreviations: HCP, healthcare professional; PwO, people with obesity.

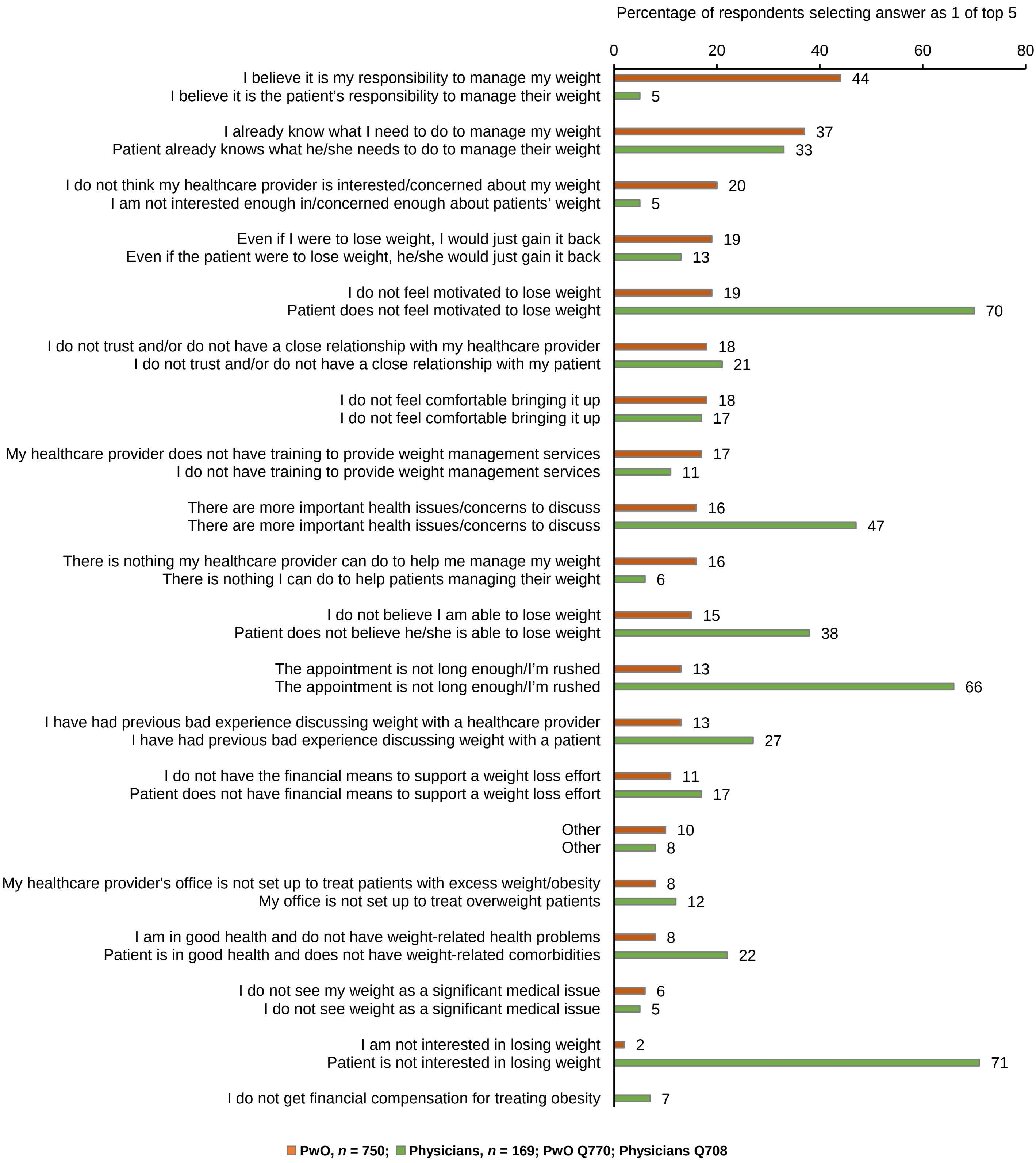

Additional file 1

FIGURE S9 Healthcare professionals with whom PwO would consider having weight management discussions
Of PwO who had not discussed weight with an HCP (physician, nurse, etc.), proportion who would consider discussing their weight with the following healthcare professionals. Abbreviations: HCP, healthcare professional; PwO, people with obesity.

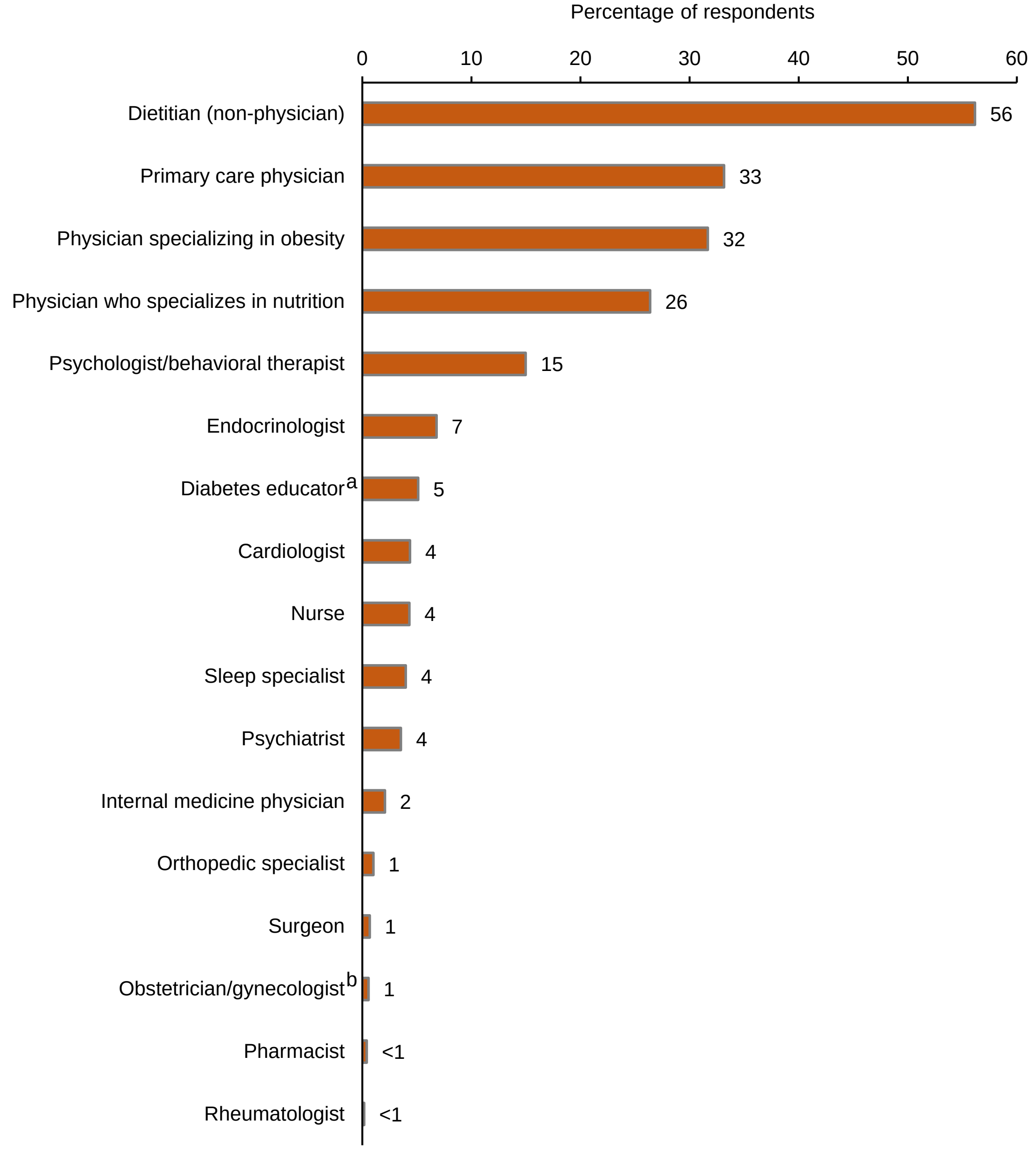

PwO who have not discussed weight with HCP, *n* = 239; Q121A.
[a]Diabetes educator not shown in some countries;
[b]Obstetrician/gynecologist not shown for males.

Additional file 1

FIGURE S10 Healthcare professionals considered most effective in helping PwO

Proportion of physicians who considered the following healthcare professionals most effective in helping PwO achieve their weight loss goals. Abbreviations: PwO, people with obesity.

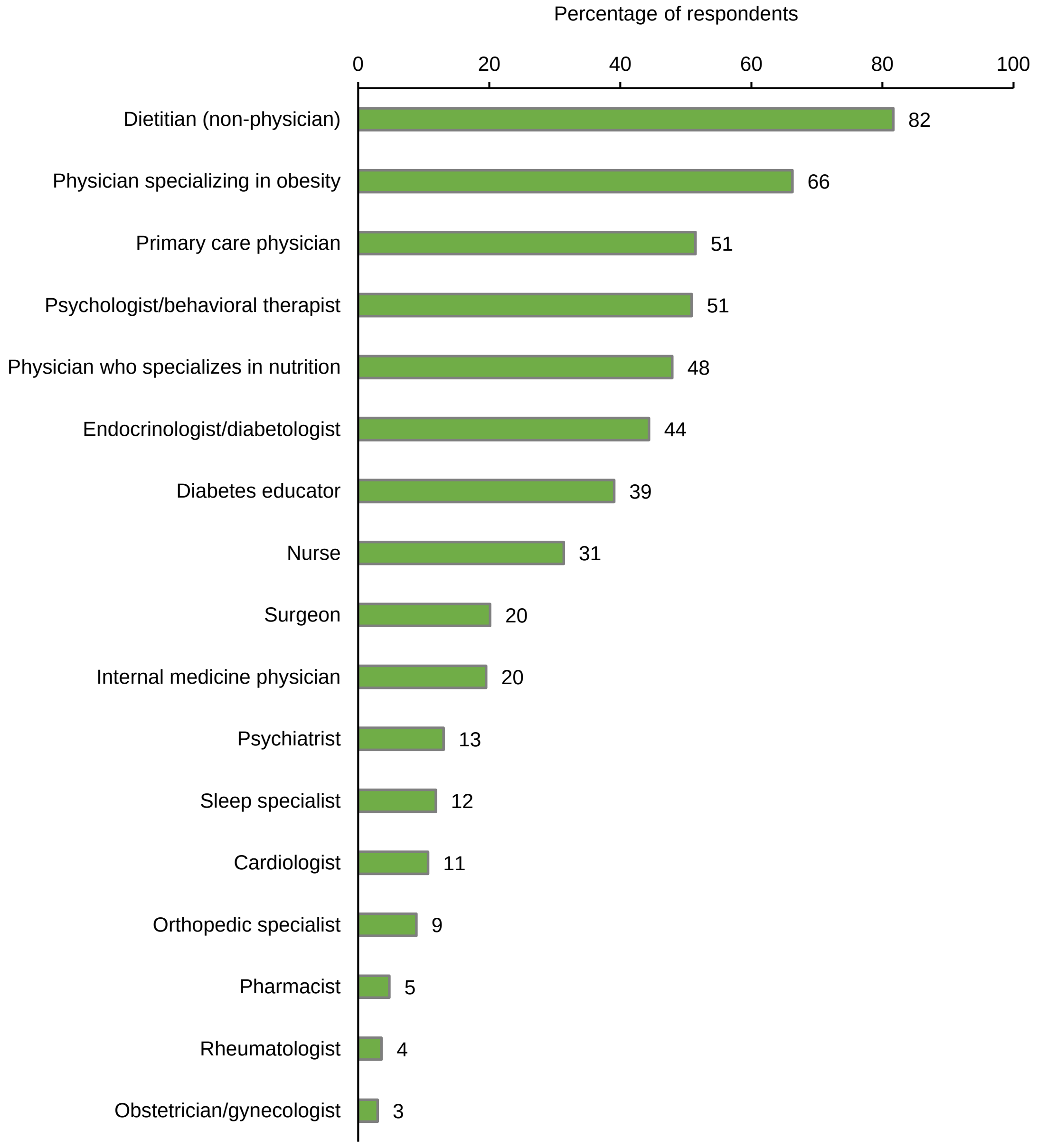

HCP, *n* = 169; Q520.
